# Transfer Potential of Plasmids Conferring Extended-Spectrum-Cephalosporin Resistance in Escherichia coli from Poultry

**DOI:** 10.1128/AEM.00654-17

**Published:** 2017-05-31

**Authors:** Solveig Sølverød Mo, Marianne Sunde, Hanna Karin Ilag, Solveig Langsrud, Even Heir

**Affiliations:** a Department of Diagnostic Services, Norwegian Veterinary Institute, Oslo, Norway; b Department of Bacteriology and Immunology, Norwegian Institute of Public Health, Oslo, Norway; c Department of Chemistry, Biotechnology and Food Science, Norwegian University of Life Sciences, Ås, Norway; d Department of Food Safety and Quality, Nofima, The Norwegian Institute for Food, Fishery and Aquaculture Research, Ås, Norway; Rutgers, The State University of New Jersey

**Keywords:** AmpC, biofilms, cephalosporin, conjugation, plasmid-mediated resistance

## Abstract

Escherichia coli strains resistant to extended-spectrum cephalosporins (ESC) are widely distributed in Norwegian broiler production, and the majority harbor transferable IncK or IncI1 plasmids carrying *bla*_CMY-2_. Persistent occurrence in broiler farms may occur through the survival of ESC-resistant E. coli strains in the farm environment, or by transfer and maintenance of resistance plasmids within a population of environmental bacteria with high survival abilities. The aim of this study was to determine the transferability of two successful *bla*_CMY-2_-carrying plasmids belonging to the incompatibility groups IncK and IncI1 into E. coli and Serratia species recipients. Initially, conjugative plasmid transfer from two E. coli donors to potential recipients was tested in an agar assay. Conjugation was further investigated for selected mating pairs in surface and planktonic assays at temperatures from 12°C to 37°C. Transfer of plasmids was observed on agar, in broth, and in biofilm at temperatures down to 25°C. The IncK plasmid was able to transfer into Serratia marcescens, and transconjugants were able to act as secondary plasmid donors to different E. coli and Serratia species recipients. All transconjugants displayed an AmpC phenotype corresponding to the acquisition of *bla*_CMY-2_. In summary, the results indicate that the IncK plasmid may transfer between E. coli and Serratia spp. under conditions relevant for broiler production.

**IMPORTANCE** Certain *bla*_CMY-2_-carrying plasmids are successful and disseminated in European broiler production. Traditionally, plasmid transferability has been studied under conditions that are optimal for bacterial growth. Plasmid transfer has previously been reported between E. coli bacteria in biofilms at 37°C and in broth at temperatures ranging from 8 to 37°C. However, intergenus transfer of *bla*_CMY-2_-carrying plasmids from E. coli to environmental bacteria in the food-processing chain has not been previously studied. We demonstrate that *bla*_CMY-2_-carrying plasmids are capable of conjugative transfer between different poultry-associated bacterial genera under conditions relevant for broiler production. Transfer to Serratia spp. and to hosts with good biofilm-forming abilities and with the potential to act as secondary plasmid donors to new hosts might contribute to the persistence of these resistance plasmids. These results contribute to increased knowledge of factors affecting the persistence of ESC resistance in broiler production and can provide a basis for improvement of routines and preventive measures.

## INTRODUCTION

During the course of the last decade, an increasing occurrence of Escherichia coli strains resistant to extended-spectrum cephalosporins (ESC) has been observed in both human and veterinary medicine (1, 2). This is concerning, as ESC are considered critically important for the treatment of human infections (3), and a reduced number of antimicrobials are available for treatment of infections caused by ESC-resistant bacteria (4). Recently, the World Health Organization included ESC-resistant Enterobacteriaceae on the top of their list of bacteria for which new antimicrobials are urgently required (5). Broilers and broiler products have been reported to be highly associated with ESC-resistant E. coli strains worldwide (1, 6). Furthermore, they have been suggested as a potential source from which humans can acquire such bacteria and/or their resistance plasmids (6–9). The Norwegian monitoring program for antimicrobial resistance in the veterinary sector (NORM-VET) has documented that ESC-resistant E. coli strains are widely distributed in Norwegian broiler production. All isolates have displayed an AmpC phenotype, mainly mediated by plasmids carrying *bla*_CMY-2_ (10–13).

Conjugative plasmids of incompatibility (Inc) groups K and I1 carrying *bla*_CMY-2_ have been associated with ESC-resistant E. coli strains from the broiler production chain in several European countries, including Norway (14–19). Also, highly similar IncK plasmids have been detected in E. coli strains of different multilocus sequence types (STs) (19). A high degree of similarity has been identified between IncK (19, 20) and IncI1 (S. S. Mo and M. Sunde, unpublished data) plasmids with *bla*_CMY-2_ isolated from broiler production in Norway and other European countries (20, 21) (Fig. S1 and S2 in the supplemental material). The findings suggest that these plasmids may be common and successful and represent plasmids endemic in European broiler production.

Import of breeding animals has been suggested as the probable source of ESC-resistant E. coli into Norwegian broiler production (22, 23), as the use of antimicrobial agents is almost absent (24–26). The number of imported batches of breeding animals positive for ESC-resistant E. coli is currently low (24, 25). Also, it has been suggested that ESC-resistant E. coli persists on broiler farms, even between production cycles (27, 28), and that the epidemiology is also affected by the transfer of ESC resistance plasmids between bacteria (29).

In broiler production, bacteria are likely to be present in suspension, on surfaces, and occasionally in biofilms. Bacteria in biofilms will form multispecies or multigenus consortia where bacterial interaction can occur under different environmental conditions and temperatures (30). Both conjugative transfer of plasmids and transduction of bacteriophages between bacteria in biofilm have been reported (31, 32). In general, conjugal transfer of plasmids has often been studied under optimal growth conditions not reflecting the conditions encountered in food production. We performed conjugation experiments on agar, in broth, and in biofilm to mimic bacterial growth on surfaces, in suspension, and in biofilm. Furthermore, all experiments were performed at three different temperatures, mimicking different parts of broiler production in addition to optimal growth conditions.

E. coli strains harboring successful plasmids with *bla*_CMY-2_ at several levels of the broiler production pyramid may facilitate horizontal spread and dissemination to other bacterial hosts in the environment, including hosts with good survival abilities. This may also play an important role in the epidemiology and maintenance of ESC resistance in broiler production. Serratia spp. are environmental bacteria commonly occurring in broiler production (33–36). In addition, they can have the ability to survive and multiply in the presence of disinfectants (37) and to be good biofilm producers (38–40). We therefore hypothesized that Serratia spp. may act as a reservoir for and secondary donor of ESC resistance plasmids. The aim of this study was to determine the transferability of two well-characterized *bla*_CMY-2_-carrying plasmids belonging to the IncK and IncI1 incompatibility groups. Transfer experiments were performed with other E. coli and Serratia spp. as recipient strains at different temperatures and under both planktonic and biofilm modes of growth, reflecting conditions relevant for the broiler production chain.

(Parts of the results of this study were presented at the 26th ECCMID conference in Amsterdam, The Netherlands, 9 to 12 April 2016.)

## RESULTS

In the initial conjugation experiments on agar, transfer of pNVI1292 carrying IncK (pNVI1292/IncK) from E. coli 1292 was observed to seven out of 14 E. coli recipients and four out of 18 Serratia species recipients (Table 1). In addition, the plasmid was transferred from Serratia marcescens and Serratia proteamaculans transconjugants to the same seven E. coli recipients. Furthermore, pNVI1292/IncK was transferred from S. marcescens transconjugants to an S. proteamaculans recipient and from the S. proteamaculans transconjugant to an S. marcescens recipient. The pNVI2798/IncI1 plasmid from E. coli 2798 was transferred to 12 out of 14 E. coli recipients. No transfer of pNVI2798/IncI1 to Serratia species recipients was observed in the initial conjugation experiment. In addition, we were unable to transform the pNVI2798/IncI1 plasmid into Serratia spp. by electroporation in subsequent control experiments. The pNVI2798/IncI1 plasmid was transformed into electrocompetent E. coli DH5α, and the pNVI1292/IncK plasmid was transformed into all three electrocompetent recipients (S. marcescens 3306 and 3307 and DH5α), confirming that the method used was reliable.

**TABLE 1:** Overview of results from initial conjugation experiments on agar

Recipient	Donor (strain, plasmid)^*a*^				
	E. coli 1292, IncK	E. coli 2798, IncI1	S. marcescens 3306 transconjugant, IncK	S. marcescens 3307 transconjugant, IncK	S. proteamaculans 5685 transconjugant, IncK
E. coli 1553	−	−	NP	NP	NP
E. coli 6154	−	+	NP	NP	NP
E. coli 706	−	+	NP	NP	NP
E. coli 7079	−	−	NP	NP	NP
E. coli 3460-5	−	+	NP	NP	NP
E. coli 6927-5	+	+	+	+	+
E. coli 1268	+	+	−	+	+
E. coli 1450	+	+	+	+	+
E. coli 1667	+	+	+	+	+
E. coli 2362	−	+	NP	NP	NP
E. coli 4922	+	+	+	+	+
E. coli 5792	+	+	+	+	+
E. coli 3064-2	+	+	+	+	+
E. coli 4064-1	−	+	NP	NP	NP
S. marcescens 2336	−	−	NP	NP	−
S. marcescens 3297	−	−	NP	NP	NP
S. marcescens 3298	−	−	NP	NP	−
S. marcescens 3299	−	−	NP	NP	−
S. marcescens 3300	−	−	NP	NP	−
S. marcescens 3301	−	−	NP	NP	−
S. marcescens 3302	−	−	NP	NP	−
S. marcescens 3303	−	−	NP	NP	−
S. marcescens 3304	−	−	NP	NP	−
S. marcescens 3305	−	−	NP	NP	−
S. marcescens 3306	+	−	NP	NP	−
S. marcescens 3307	+	−	NP	NP	+
S. marcescens 3308	−	−	NP	NP	−
S. marcescens 3309	+	−	+	+	−
Serratia sp. 3612	−	−	NP	NP	NP
S. liquefaciens 5676	−	−	−	−	−
S. proteamaculans 5682	−	−	NP	NP	NP
S. proteamaculans 5685	+	−	+	+	NP

a +, confirmed plasmid transfer; −, no observed plasmid transfer; NP, not performed.

PCR screening for the IncK and IncI1 replicons showed that none of the recipient strains carried IncK or IncI1 plasmids prior to the experiments.

Seven mating pairs were selected and subjected to extended conjugation experiments under various temperatures and modes of bacterial growth (Tables 2 and 3). In the extended conjugation experiments, plasmid transfer was observed on agar, in broth, and in biofilm at 25, 30, and 37°C (Tables 2 and 3). No plasmid transfer was observed at 12°C (data not shown).

**TABLE 2:** Overview of results from extended conjugation experiments on agar and in broth

Mating pair (donor→recipient)	Mating time (h)	Conjugation result at/in^*a*^:			
		25°C		30/37°C^*b*^	
		Agar	Broth^*c*^	Agar	Broth^*c*^
E. coli 1292(IncK)→E. coli 6927-5	4	NA	NA	+	+
	24	+	+	+	+
	48	+	+	+	+
E. coli 2798(IncI1)→E. coli 6927-5	4	NA	NA	+	+
	24	−	+	+	+
	48	−	+	+	+
E. coli 1292(IncK)→S. marcescens 3306	4	NA	NA	−	+
	24	+	−	−	+
	48	−	−	−	+
E. coli 1292(IncK)→S. marcescens 3307	4	NA	NA	+	+
	24	+	+	+	+
	48	+	+	+	+
S. marcescens 3306 transconjugant (IncK)→E. coli 6927-5	4	NA	NA	+	+
	24	+	+	+	+
	48	+	+	+	+
S. marcescens 3307 transconjugant (IncK)→E. coli 6927-5	4	NA	NA	+	+
	24	+	+	+	+
	48	+	+	+	+
S. marcescens 3307 transconjugant (IncK)→S. proteamaculans 5685	4	NA	NA	+	+
	24	−	−	+	+
	48	−	+	+	+

a +, confirmed transfer of plasmid carrying *bla*_CMY-2_; −, no observed transfer of *bla*_CMY-2_-carrying plasmid; NA, not applicable.

b 30°C was applied for all matings involving Serratia spp., while 37°C was applied for matings involving E. coli only.

c Transconjugant detection limit, 10 CFU/ml.

**TABLE 3:** Overview of maximum transfer frequencies for different mating pairs and incubation times for conjugation experiments in biofilm

Mating pair (donor→recipient)	Mating time (h)	Transfer frequency at^*a*^:	
		25°C	30/37°C^*b*^
E. coli 1292(IncK)→E. coli 6927-5	4	NA	NTD
	24	3 × 10^−6^	5 × 10^−4^
	48	3 × 10^−6^	8 × 10^−2^
E. coli 2798(IncI1)→E. coli 6927-5	4	NA	4 × 10^−5^
	24	NTD	4 × 10^−3^
	48	NTD	2 × 10^−2^
E. coli 1292(IncK)→S. marcescens 3306	4	NA	NTD
	24	NTD	NTD
	48	NTD	NTD
E. coli 1292(IncK)→S. marcescens 3307	4	NA	NTD
	24	NTD	NTD
	48	NTD	7 × 10^−6^
S. marcescens 3306 transconjugant (IncK)→E. coli 6927-5	4	NA	9 × 10^−6^
	24	2 × 10^−6^	2 × 10^−5^
	48	NTD	3 × 10^−4^
S. marcescens 3307 transconjugant (IncK)→E. coli 6927-5	4	NA	1 × 10^−5^
	24	2 × 10^−5^	6 × 10^−4^
	48	9 × 10^−5^	2 × 10^−3^
S. marcescens 3307 transconjugant (IncK)→S. proteamaculans 5685	4	NA	NTD
	24	NTD	NTD
	48	NTD	5 × 10^−7^

a The transfer frequencies are calculated as the total number of transconjugants (T) divided by the total number of recipients (R). NTD, no transconjugants detected, transfer frequency of <5 × 10^−7^; NA, not applicable.

b 30°C was applied for all matings involving Serratia spp., while 37°C was applied for matings involving E. coli only.

**FIG 1:**
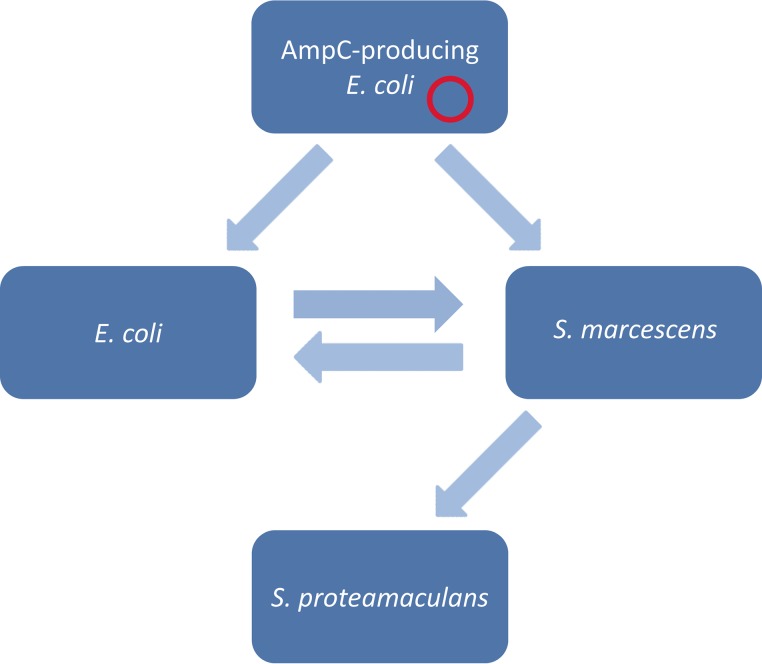
Schematic overview of confirmed routes of conjugative transfer in biofilm for the pNVI1292/IncK plasmid harboring *bla*_CMY-2_ commonly found in E. coli strains resistant to extended-spectrum cephalosporins isolated from retail chicken meat. The red circle symbolizes the IncK plasmid in the initial E. coli host. The arrows indicate the routes of transfer confirmed in this study.

When transfer of plasmids occurred in the biofilm experiments, the transfer frequencies of the pNVI1292/IncK plasmid (T/R) ranged from 5 × 10^−7^ to 8 × 10^−2^. The highest frequencies were observed between E. coli-E. coli mating pairs, ranging from 3 × 10^−6^ to 8 × 10^−2^ (Table 3). Furthermore, the pNVI1292/IncK plasmid was transferred from E. coli to S. marcescens, from the S. marcescens transconjugant to an E. coli recipient, and from the S. marcescens transconjugant to an S. proteamaculans recipient in biofilm. The confirmed routes for horizontal transfer of the pNVI1292/IncK plasmid in biofilm are illustrated in Fig. 1. The transfer frequencies observed for the pNVI2798/IncI1 plasmid ranged from 4 × 10^−5^ to 2 × 10^−2^. The pNVI2798/IncI1 plasmid was not subjected to conjugation experiments with S. marcescens as a recipient in biofilm on steel coupons, as no transfer was observed after the initial conjugation experiments on agar. In general, the transfer frequencies were higher at 30°C/37°C than at 25°C.

None of the recipient strains were resistant to any beta-lactam antimicrobials prior to the conjugation experiments. All transconjugants displayed a beta-lactam resistance profile corresponding to an AmpC phenotype after acquisition of either the pNVI1292/IncK or pNVI2798/IncI1 plasmid (Table 4). No additional determinants conferring resistance to the antimicrobials tested were transferred with the plasmids (Table S2).

**TABLE 4:** MICs of beta-lactam antimicrobials for recipient strains before acquisition of pNVI1292/IncK or pNVI2798/IncI1 plasmid, and for transconjugants after acquisition of pNVI1292/IncK and pNVI2798/IncI1 plasmids

Recipient/transconjugant strain (plasmid)	MIC (epidemiological cutoff value for antimicrobial) (mg/liter)^*a*^									
	FOX (8)	ETP (0.06)	IMI (0.5)	MERO (0.12)	TAZ (0.5)	FEP (0.12)	F/C (0.25/4)	T/C (0.5/4)	FOT (0.25)	TRM (NA)
E. coli 6927-5	4	≤0.015	≤0.12	≤0.03	≤0.25	≤0.06	≤0.06/4	≤0.012/4	≤0.25	4
E. coli 6927-5(IncK)	32	0.03	≤0.12	≤0.03	8	0.12	4/4	4/4	4	4
E. coli 6927-5(IncI1)	64	0.03	≤0.12	≤0.03	16	0.25	4/4	8/4	8	4
S. marcescens 3306	16	≤0.015	0.5	0.06	≤0.25	≤0.06	≤0.06/4	0.5/4	≤0.25	8
S. marcescens 3306(IncK)	32	0.03	0.5	0.06	8	0.25	4/4	8/4	4	8
S. marcescens 3307	8	≤0.015	0.5	≤0.03	≤0.25	≤0.06	≤0.06/4	≤0.12/4	≤0.25	8
S. marcescens 3307(IncK)	32	0.06	0.5	0.6	8	0.25	8/4	8/4	4	8
S. proteamaculans 5685	4	≤0.015	0.25	≤0.03	≤0.25	≤0.06	≤0.06/4	≤0.12/4	≤0.25	4
S. proteamaculans 5685(IncK)	64	≤0.015	0.25	≤0.03	2	0.12	4/4	1/4	8	4

a FOX, cefoxitin; ETP, ertapenem; IMI, imipenem; MERO, meropenem; TAZ, ceftazidime; FEP, cefepime; F/C, cefotaxime-clavulanic acid; T/C, ceftazidime-clavulanic acid; FOT, cefotaxime; TRM, temocillin; NA, not available.

## DISCUSSION

Conjugative IncK and IncI1 plasmids harboring *bla*_CMY-2_ are commonly occurring in ESC-resistant E. coli strains in European broiler production (14–19). To our knowledge, this is the first description of the transfer of these plasmids between E. coli and Serratia spp. under conditions relevant in the broiler production chain. In food-processing environments, bacteria will be present and grow on the surfaces of equipment and environments where biofilm formation and interaction between organisms are likely to occur. Likewise, the present bacteria will also be suspended in liquids, both during process and postprocess cleaning and disinfection. Conjugation experiments were therefore performed on surfaces, in biofilms on stainless steel, and in suspension at various temperatures. Transfer of plasmids was observed in conjugation experiments on agar, in broth, and in biofilm at 25°C and 30/37°C. In general, we observed higher transfer of plasmids at 30/37°C than at 25°C, which is in correspondence with previous findings (41). The fact that horizontal transfer of plasmids occurs at 25°C might indicate a possible relevance in parts of the production chain with higher temperatures, such as inside the broiler house. Low temperatures have been reported to decrease the conjugal transfer of plasmids in broth matings (41). No plasmid transfer was observed at 12°C. This indicates that conjugal transfer of resistance plasmids is a limited problem in the parts of food production where low temperatures are applied. Low temperatures in processing units may thus reduce or inhibit horizontal transfer of plasmids as well as hamper microbial growth. On the other hand, transfer of plasmids has previously been reported to take place at 8°C (41). Therefore, we cannot exclude the possibility that the plasmids can be transferred to and maintained within hosts that can facilitate further dissemination under growth conditions that are suboptimal for mesophilic E. coli but more optimal for psychrophilic bacteria.

Bacteria in biofilm can have an increased ability to survive cleaning and disinfection (42–45), possibly serving as a reservoir for recontamination of environments and foods after disinfection. All recipients included in the biofilm experiments showed moderate to good biofilm-forming abilities on stainless steel coupons at 12°C and 25°C (data not shown). Thus, it is reasonable to suggest that survival of ESC-resistant bacteria in biofilm, together with conjugative transfer of plasmids, is also a part of the puzzle when it comes to the maintenance and dissemination of ESC-resistant bacteria and plasmids.

We have demonstrated the ability of the pNVI1292/IncK plasmid to transfer within and between genera in the Enterobacteriaceae family. In addition, we have shown that transconjugants can act as secondary plasmid donors. The relatively high transfer frequencies observed from S. marcescens transconjugants to E. coli recipients indicate the ability of environmental bacteria to be efficient contributors in the dissemination of IncK resistance plasmids. The ability of Serratia spp. to acquire and harbor such resistance plasmids and act as secondary plasmid donors support the hypothesis that Serratia spp. can represent a reservoir for plasmid-mediated ESC resistance.

The pNVI1292/IncK and pNVI2798/IncI1 plasmids differed in their ability to transfer to or replicate within different Enterobacteriaceae hosts. Both plasmids were transferred to E. coli recipients, but no transfer of the pNVI2798/IncI1 plasmid to Serratia species recipients was observed in any of our conjugation or transformation experiments. None of the recipient strains were shown to harbor other IncI1 plasmids and should therefore be able to receive the pNVI2798/IncI1 plasmid. Based on these results, it was not possible to determine if the pNVI2798/IncI1 plasmid is unable to transfer into Serratia species hosts or if it is unable to replicate within Serratia species hosts. As only a limited number of strains and mating combinations were investigated, transfer of the pNVI2798/IncI1 plasmid or related plasmids to Serratia spp. cannot be excluded. Further experiments should be performed in order to determine whether pNVI1292/IncK has a higher ability of transfer to and propagate in different bacterial hosts than pNVI2798/IncI1.

In the biofilm experiments, the number of transconjugants increased with time, which has also been described previously (46). However, with the method used here, it is not possible to determine if (i) this was due to an increased number of conjugative transfers from donor to recipient, (ii) transconjugants started to act as donors as well, (iii) transconjugant strains reproduced in the biofilm, or (iv) a combination of the three. Furthermore, it is not possible to exclude that the observed plasmid transfer in biofilm may actually have occurred in the broth in which the steel coupon with the biofilm was submerged. Recipient cells from the biofilm might have detached and entered the broth, received the plasmid from donor cells in the broth, and thereafter reattached to the biofilm. Surprisingly, transconjugants were observed after 24 h but not after 48 h for some mating pairs. This is probably due to methodological limitations, with the number of transconjugants being around the detection limit, causing inconsistent results for consecutive samplings. Another explanation could be that the plasmid was lost after 48 h. This seems less likely, as plasmid stability systems are present on both plasmids (19, 21).

In this study, we have demonstrated the ability of conjugative plasmids of poultry origin encoding ESC resistance to transfer into different E. coli and Serratia spp. both in suspension and on surfaces at different temperatures. Transfer occurred under suboptimal growth conditions and in biofilm, underlining the potential for horizontal transfer of these resistance plasmids. In food production, vertical spread of antimicrobial resistance through clonal dissemination is likely to represent a higher burden than the horizontal spread of resistance determinants between bacterial clones and/or genera. However, transfer to environmental Enterobacteriaceae or other residential bacteria with good survival abilities (e.g., growth at low temperature, biofilm formation, and increased tolerance to environmental conditions, such as cleaning and disinfection) and with potential to act as secondary plasmid donors to new hosts may contribute to maintenance of the resistance plasmids through the food chain. The results also indicate that low temperatures may contribute to a decrease in plasmid transfer and hamper microbial growth. Further research on the occurrence of ESC resistance in environmental bacteria and transfer of plasmids in models simulating relevant conditions is necessary to determine the importance of the environmental microbiota and environmental conditions for maintenance and dissemination of ESC resistance in broiler production.

## MATERIALS AND METHODS

### Bacterial isolates.

Plasmid donor isolates used included E. coli 2012-01-1292 (designated E. coli 1292) carrying a recently characterized IncK plasmid (pNVI1292/IncK, accession no. KU312044, Fig. S1) (19) and E. coli 2012-01-2798 (designated E. coli 2798) carrying an IncI1 plasmid (pNVI2798/IncI1, Fig. S2). Both donor strains harbored *bla*_CMY-2_ and originated from domestically produced retail chicken meat collected in 2012 as part of the NORM-VET program (11). The plasmids did not harbor additional resistance genes. Potential recipient strains included a selection of E. coli from broiler feces and retail chicken meat (*n* = 14) displaying resistance to nalidixic acid (Nal^r^), and Serratia spp. from food-processing units and retail chicken meat (*n* = 18). The Serratia species isolates were rifampin resistant (Rif^r^) (*n* = 15) or made Rif^r^ (*n* = 3) by subculturing in broth with increasing Rif concentration, as previously described (47). None of the recipient strains displayed resistance to any beta-lactam antimicrobials (data not shown). All included recipient strains were subjected to PCR targeting the IncI1 and IncK replicons to ensure that they did not harbor other plasmids of these incompatibility groups (48). An overview of the included isolates and their characteristics is given in Table 5.

**TABLE 5:** All isolates included in the study

Isolate ID (strain type)^*a*^	Origin	Resistance profile^*b*^	MIC (mg/liter)	Phylotype^*c*^
E. coli 1292(IncK) (D)	Retail chicken meat	Ctx^r^	>2	D
E. coli 2798(IncI1) (D)	Retail chicken meat	Ctx^r^	>2	A
E. coli 1553 (R)	Retail chicken meat	Nal^r^	128	ND
E. coli 6154 (R)	Retail chicken meat	Nal^r^	>128	ND
E. coli 706 (R)	Retail chicken meat	Nal^r^	>128	ND
E. coli 7079 (R)	Retail chicken meat	Nal^r^	32	ND
E. coli 3460-5 (R)	Fecal flora of healthy broiler	Nal^r^	>128	ND
E. coli 6927-5 (R)^*d*^	Fecal flora of healthy broiler	Nal^r^	>128	B1
E. coli 1268 (R)	Fecal flora of healthy broiler	Nal^r^	32	B1
E. coli 1450 (R)	Fecal flora of healthy broiler	Nal^r^	64	D
E. coli 1667 (R)	Fecal flora of healthy broiler	Nal^r^	32	D
E. coli 2362 (R)	Fecal flora of healthy broiler	Nal^r^	64	D
E. coli 4922 (R)	Fecal flora of healthy broiler	Nal^r^	64	D
E. coli 5792 (R)	Fecal flora of healthy broiler	Nal^r^	128	D
E. coli 3064-2 (R)	Fecal flora of healthy broiler	Nal^r^	128	A
E. coli 4064-1 (R)	Fecal flora of healthy broiler	Nal^r^	128	ND
S. marcescens 2336 (R)	Disinfecting footbath on dairy plant	Rif^r^	≥16	NA
S. marcescens 3297 (R)	Disinfecting footbath on dairy plant	Rif^r^	≥16	NA
S. marcescens 3298 (R)	Disinfecting footbath on dairy plant	Rif^r^	≥16	NA
S. marcescens 3299 (R)	Disinfecting footbath on dairy plant	Rif^r^	≥16	NA
S. marcescens 3300 (R)	Disinfecting footbath on dairy plant	Rif^r^	≥16	NA
S. marcescens 3301 (R)	Disinfecting footbath on dairy plant	Rif^r^	≥16	NA
S. marcescens 3302 (R)	Disinfecting footbath on dairy plant	Rif^r^	≥16	NA
S. marcescens 3303 (R)	Disinfecting footbath on dairy plant	Rif^r^	≥16	NA
S. marcescens 3304 (R)	Disinfecting footbath on dairy plant	Rif^r^	≥16	NA
S. marcescens 3305 (R)	Disinfecting footbath on dairy plant	Rif^r^	≥16	NA
S. marcescens 3306 (R)^*d*^	Disinfecting footbath on dairy plant	Rif^r^	≥16	NA
S. marcescens 3307 (R)^*d*^	Disinfecting footbath on dairy plant	Rif^r^	≥16	NA
S. marcescens 3308 (R)	Disinfecting footbath on dairy plant	Rif^r^	≥16	NA
S. marcescens 3309 (R)	Disinfecting footbath on dairy plant	Rif^r^	≥100	NA
Serratia sp. 3612 (R)	Slaughterhouse	Rif^r^	≥16	NA
Serratia liquefaciens 5676 (R)	Retail chicken meat	Rif^r^	≥100	NA
S. proteamaculans 5682 (R)	Retail chicken meat	Rif^r^	≥16	NA
S. proteamaculans 5685 (R)^*d*^	Retail chicken meat	Rif^r^	≥100	NA

a ID, identification; D, donor strain; R, recipient strain.

b Ctx^r^, cefotaxime resistant; Nal^r^, nalidixic acid resistant; Rif^r^, rifampin resistant.

c ND, not determined; NA, not applicable.

d Isolates included in extended conjugation experiment.

### Preculturing of donor and recipient strains.

Precultures of donor and recipient strains used in the initial screening experiments were grown on tryptone soy agar (TSA) plates (Oxoid Ltd., Basingstoke, England) at 30°C (Serratia spp.) or 37°C (E. coli) overnight. In the extended conjugation experiments, precultures for strains used in conjugation on agar were grown on TSA at 25°C for 3 days. Before mating was performed, the agar plates were incubated at the temperature used in the subsequent experiment for 1 h. Precultures of donor and recipient strains used in experiments in broth and donor strains used in the biofilm experiments at 25°C, 30°C, and 37°C were grown separately in Luria-Bertani (LB) broth (Oxoid Ltd.) (broth mating) or LB without NaCl (biofilm experiments) at 25°C for 3 days. Thereafter, they were incubated at the temperature used in the subsequent experiment for 1 h before broth mating was performed. Precultures used in experiments at 12°C were incubated at 12°C for 3 days. Recipient strains used in the biofilm experiments were grown in LB without NaCl at 30°C overnight.

### Conjugation experiments.

We used a two-step strategy for investigation of plasmid transfer. This included an initial screening using a simple agar assay, followed by extended transfer experiments with selected mating pairs performed under various conditions.

In the initial experiments, the two E. coli donor strains were mated with 32 recipient strains using conjugation experiments on agar to identify mating pairs where plasmids were transferred. In addition, we investigated if Serratia species transconjugants could act as secondary plasmid donors. Two S. marcescens transconjugants were mated with E. coli (*n* = 7) and Serratia spp. (*n* = 3), and one S. proteamaculans transconjugant was mated with E. coli (*n* = 7) and Serratia spp. (*n* = 16). The conjugation experiments on agar were carried out as follows: one colony each of the recipient and the donor were mixed on a TSA plate and incubated at 30°C. Matings were sampled after 4, 24, and 48 h by swiping a loop through the colonies. Samples were plated directly on Mueller-Hinton (MH) agar (Difco, Sparks, MD, USA) supplemented with two different antimicrobials used to select for transconjugant strains. The antimicrobials and concentrations used to select for transconjugant strains are presented in Table S1.

Extended conjugation experiments were carried out with selected mating pairs on agar surface, in broth, and in biofilm under various temperatures in order to mimic conditions relevant for broiler production. Mating pairs were selected on the basis of the recipients' ability to receive the plasmid in the initial screening experiments. Conjugation experiments in biofilm were performed in triplicate. All experiments were performed at 12°C, 25°C and 30°C (Serratia spp.) or 37°C (E. coli), and sampling was performed after 4 h (30°C and 37°C), 24 h, and 48 h (all matings).

### Conjugation on agar.

Conjugation experiments on agar were performed as described above.

### Conjugation in broth.

Conjugation in broth was conducted as previously described (49). Briefly, 1 ml of the donor preculture and 1 ml of the recipient preculture were mixed in 4 ml of fresh LB broth. Sampling was performed by plating 100 μl of the broth on MH agar supplemented with two different antimicrobials used to select for transconjugant strains.

### Conjugation in biofilm.

Biofilms of each recipient strain were established on autoclaved coupons of stainless steel (AISI 304), as previously described (50). Thereafter, the tubes were incubated at 25°C, 30°C (S. marcescens), or 37°C (E. coli) for 3 days or at 12°C for 5 days. The steel coupons with established biofilms were washed with peptone water to remove loosely attached cells and then transferred to tubes with cultures of the donor strain (31). Sampling of biofilms included harvesting the whole biofilm. The steel coupon was rinsed in peptone water and transferred to a glass tube with 15 ml of peptone water. The biofilm was detached by scraping with a swab, followed by 15 min of sonication at 42 kHz at 25°C. One milliliter of the samples was diluted 10-fold, and 100 μl of the dilution series was plated out on three different MH agar plates supplemented with different antimicrobials in order to quantify number of donors, recipients, and transconjugants in the sample. The transfer frequencies of plasmids in biofilms were calculated as number of transconjugants/number of recipients (T/R) (49, 51).

All MH agar plates were incubated at 30°C (Serratia spp.) or 37°C (E. coli) for 24 h.

### Transformation experiments.

Transformation was performed to investigate if it was possible to introduce the pNVI2798/IncI1 plasmid into electrocompetent S. marcescens hosts (3306 and 3307). In addition, transformation of the pNVI1298/IncK plasmid was included as a positive control under the experimental conditions applied. Plasmid DNA was purified as described by Engebrecht et al. (52), with some modifications. After completing step 5 in the original protocol, the tubes were centrifuged for five min at 14,000 rpm, and 270 μl of the supernatant was added to Eppendorf tubes containing 270 μl of 5 M LiCl. The mixture was vortexed and placed at −20°C for 10 min. Subsequently, the tubes were centrifuged for 5 min at 14,000 rpm, and 500 μl of the supernatant was transferred to a new tube containing 1,000 μl of ethyl alcohol (EtOH). The mixture was left at −20°C for 10 min and subjected to centrifugation for 10 min at 14,000 rpm before discarding the supernatant. Then, 200 μl of 70% EtOH was added, and the tubes were centrifuged for 10 min at 13,000 rpm. After discarding the supernatant, the pellet was resuspended in 30 μl of Tris-EDTA (TE) buffer. S. marcescens 3306 and 3307 and E. coli DH5α (CCUG 32825) were made electrocompetent, as previously described (53). However, three washing steps were performed. Also, the pellet was resuspended in Milli Q water instead of transformation buffer or DnD solution and used immediately in transformation experiments. Electroporation was performed on 5 μl of plasmid DNA mixed with 50 μl of competent cells as follows: 1.25 kV/cm, 200 Ω, 25 microfarad.

Transformants were selected on LB agar supplemented with 1 mg/liter cefotaxime. The transformation experiments were performed in triplicate.

### Confirmation of transconjugants and transformants.

Donor and recipient strains of different genera and species (E. coli-S. marcescens matings and S. marcescens-S. proteamaculans matings) were differentiated by the use of matrix-assisted laser desorption ionization–time of flight mass spectrometry (MALDI-TOF MS; Bruker Daltonics). Phylotyping (54) was used for differentiation of mating pairs with E. coli as both the donor and recipient (Table 5). Transconjugants and transformants were confirmed by real-time PCR detection of *bla*_CMY-2_ using previously published primers and probe (55). Positive and negative controls were included in each run.

### Antimicrobial susceptibility testing.

Recipients and transconjugants were subjected to antimicrobial susceptibility testing to determine the MICs for a standard panel of antimicrobials and an extended panel of relevant beta-lactam antimicrobials by broth microdilution (EUVSEC and EUVSEC2, Sensititre TREK; Thermo Scientific). E. coli ATCC 25922 was included as a quality control.

## Supplementary Material

Supplemental material
