# Supplementary material for: Transfer Potential of Plasmids Conferring Extended-Spectrum-Cephalosporin Resistance in Escherichia coli from Poultry
Source: Appl Environ Microbiol. 2017 May 31;83(12):e00654-17. doi: 10.1128/AEM.00654-17 (PMC5452821; doi:10.1128/AEM.00654-17)
Supplement: Supplemental material [file supp_83_12_e00654-17__index.html]

Supplemental material 

# Transfer Potential of Plasmids Conferring Extended-Spectrum-Cephalosporin Resistance in Escherichia coli from Poultry

## Supplemental material

- Supplemental file 1 -

  Highly similar IncK (Fig. S1) and IncI1 (Fig. S2) plasmids from the European broiler production, antimicrobials and concentrations used in selective Mueller-Hinton agar plates used to identify transconjugant strains in conjugation experiments (Table S1), and MICs.

  PDF, 465K
