# Supplementary material for: Transfer Potential of Plasmids Conferring Extended-Spectrum-Cephalosporin Resistance in Escherichia coli from Poultry
Source: Appl Environ Microbiol. 2017 May 31;83(12):e00654-17. doi: 10.1128/AEM.00654-17 (PMC5452821; doi:10.1128/AEM.00654-17)
Supplement: Supplemental material [file AEM.00654-17_zam999117885s1.pdf]

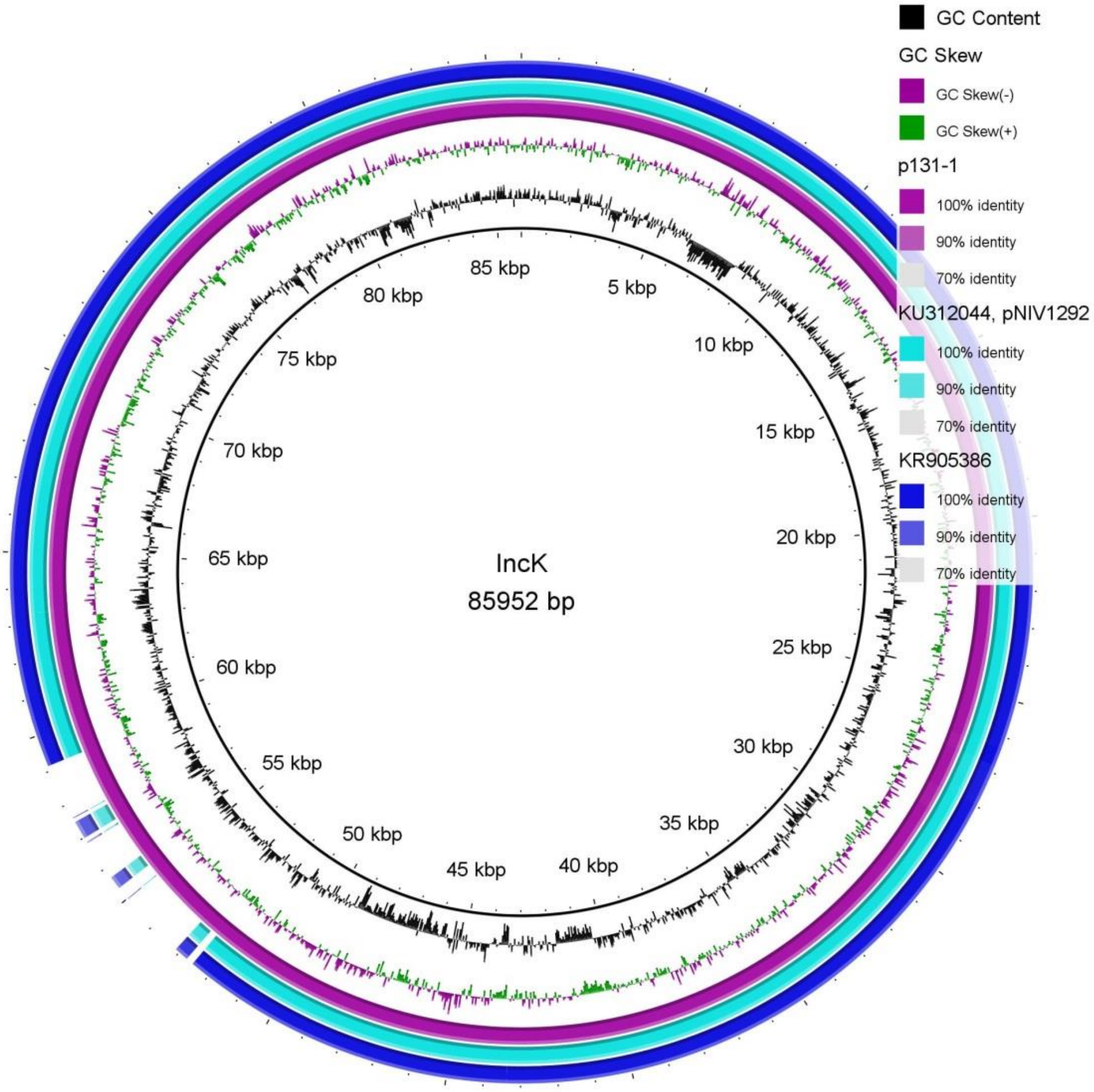

Figure S1: Blast ring image showing highly similar IncK plasmids from the European broiler production. An IncK plasmid from the Netherlands (accession number NZ_JXMX01000007.1) is used as reference, and one plasmid from Denmark (p131-1), one from Norway (KU312044, pNVI1292/IncK) and one from Switzerland (accession number KR905386.1) is compared to this plasmid.

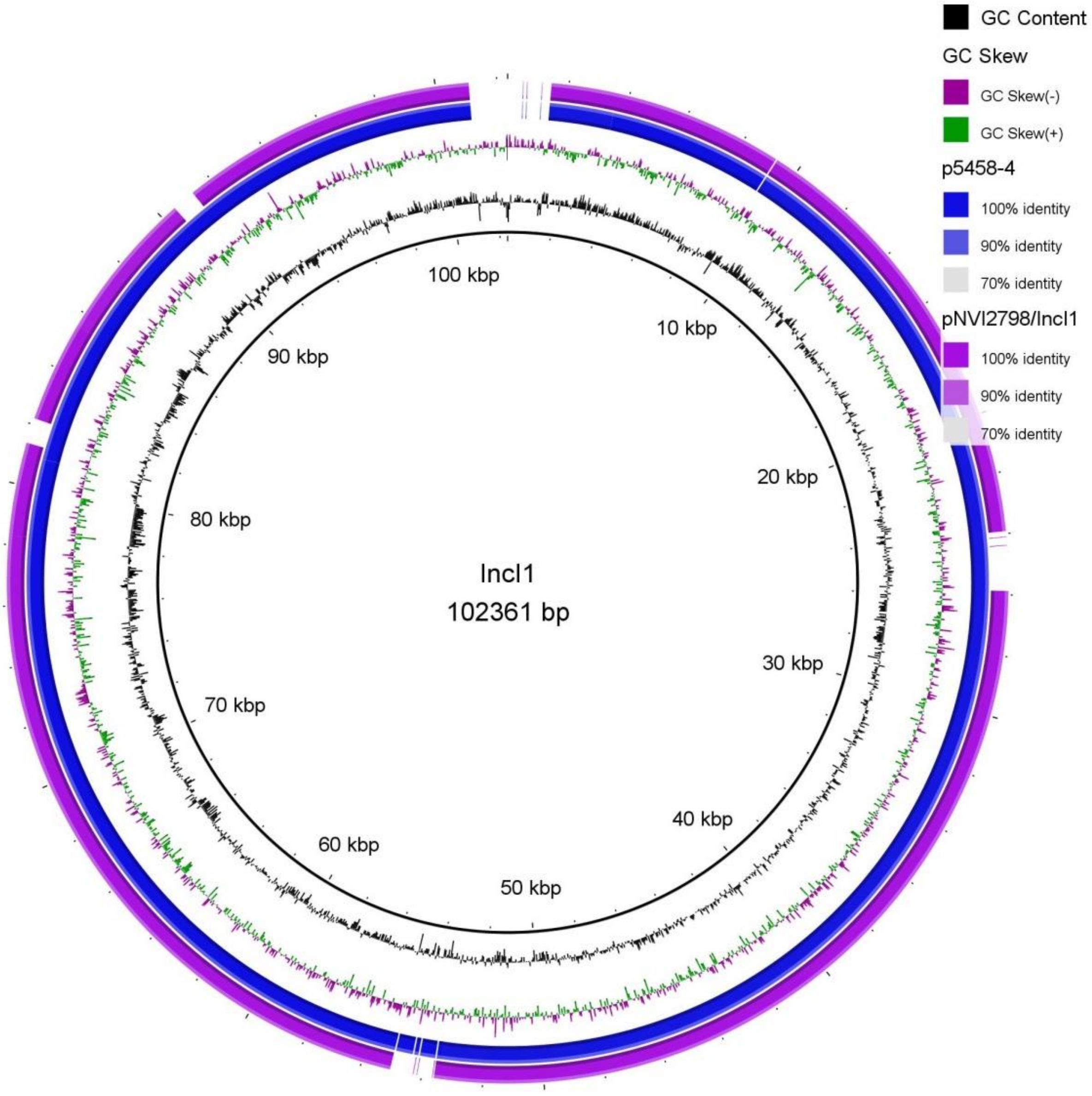

Figure S2: Blast ring image showing highly similar IncI1 plasmids from the European broiler production. ESBL-355 (accession number 53168488) from the Netherlands is used as reference, and one IncI1 plasmid from Denmark (p5458-4) and one from Norway (pNVI2798/IncI1) is compared to this plasmid.

Table S1. Antimicrobials and concentrations used in selective Mueller Hinton agar plates used to identify transconjugant strains in conjugation experiments.

Donor phenotype (MIC)	Recipient phenotype (MIC)	Antimicrobial concentrations in plates used to detect transconjugants
Ctx^R (>2 mg/L)	Nal^R (≥32 mg/L)	0,5 mg/L Ctx + 20 mg/L Nal
Ctx^R (>2 mg/L)	Rif^R (≥16 mg/L)	0,5 mg/L Ctx + 16 mg/L Rif
Ctx^R (>2 mg/L)	Rif^R (≥100 mg/L)	0,5 mg/L Ctx + 100 mg/L Rif
CtxR (> 2 mg/L), Rif^R (≥16 mg/L)	Rif^R (≥100 mg/L)	1 mg/L Ctx + 100 mg/L Rif

Ctx=Cefotaxime, Rif=Rifampicin, Nal= Nalidixic acid, Ctx^R=Cefotaxime resistant, Nal^R=Nalidixic acid resistant, Rif^R=Rifampicin resistant, MIC=minimum inhibitory concentration

Table S2. Minimum inhibitory concentrations (MICs) of antimicrobials for recipient strains before acquisition of pNVI1292/IncK or pNVI2798/IncI1 plasmids, and for transconjugants after acquisition of pNVI1292/IncK and pNVI2798/IncI1 plasmids.

Recipient and transconjugant strains (plasmid)	Antimicrobial (epidemiological cut-off value, mg/L)										
	SMX (64)	TMP (2)	CIP (0.064)	TET (8)	AZI (NA)	NAL (16)	CHL (16)	TGC (0.5)	COL (2)	AMP (8)	GEN (2)
E. coli 6927-5	>1024	>32	0.25	≤2	4	>128	≤8	≤0.25	≤1	>64	1
E. coli 6927-5 (IncK)	>1024	>32	0.25	≤2	4	>128	≤8	≤0.25	≤1	>64	1
E. coli 6927-5 (IncI1)	>1024	>32	0.5	≤2	4	>128	≤8	≤0.25	≤1	>64	1
S. marcescens 3306	≤8	≤0.25	0.06	64	16	≤4	≤8	0.5	>16	16	≤0.5
S. marcescens 3306 (IncK)	≤8	≤0.25	0.06	32	8	≤4	≤8	0.5	>16	>64	1
S. marcescens 3307	≤8	≤0.25	0.06	64	32	≤4	≤8	0.5	>16	8	≤0.5
S. marcescens 3307 (IncK)	≤8	≤0.25	0.06	64	32	≤4	≤8	0.5	>16	>64	≤0.5
S. proteamaculans 5685	≤8	≤0.25	≤0.015	≤2	4	≤4	≤8	≤0.25	16	2	≤0.5

S. proteamaculans 5685 (IncK)	≤8	≤0.25	≤0.015	≤2	≤2	≤4	≤8	≤0.25	16	64	≤0.5

SMX: sulfamethoxazole, TMP: trimethoprim, CIP: ciprofloxacin, TET: tetacycline, AZI: azitromycin, NAL: nalidixic acid, CHL: chloramphenicol, TGC: tigecyclin, COL: colistin, AMP: ampicillin, GEN: gentamycin, NA: not available
